# Retraction: Influence of the use of various imaging units and projections on the radiation dose received by children during chest digital radiography

**DOI:** 10.1371/journal.pone.0324184

**Published:** 2025-05-09

**Authors:** The PLOS One Editors

The *PLOS One* Editors retract this article [1] due to concerns about compromised peer review. We regret that the issues were not identified prior to the article’s publication.

All authors did not agree with the retraction.

## References

[1] Xu H, Huang K, Liu B, Cai J, Zheng H, Zheng H, et al. Influence of the use of various imaging units and projections on the radiation dose received by children during chest digital radiography. PLoS One. 2021;16(8):e0255749. doi: 10.1371/journal.pone.0255749 34352022 PMC8341633

